# Shotgun metagenomic sequence data from milk and fecal samples of dairy cattle in Thailand

**DOI:** 10.1128/mra.00564-26

**Published:** 2026-08-14

**Authors:** Songphon Buddhasiri, Tawatchai Singhla, Sahaphop Pengpanun, Thanaporn Eiamsam-Ang, Parameth Thiennimitr

**Affiliations:** 1 Veterinary Public Health and Food Safety Centre for Asia Pacific, Faculty of Veterinary Medicine, Chiang Mai University 26682, Chiang Mai, Thailand; 2 Faculty of Veterinary Medicine, Chiang Mai University 26682, Chiang Mai, Thailand; 3 Department of Microbiology, Faculty of Medicine, Chiang Mai University 26682, Chiang Mai, Thailand; University of Maryland School of Medicine, Baltimore, Maryland, USA

**Keywords:** shotgun metagenomics, dairy cattle, milk microbiome, fecal microbiome, resistome, gut microbiome

## Abstract

We report shotgun metagenomic sequence data from milk and fecal samples of dairy cattle in Thailand. This data set captures microbial genetic profiles from mammary- and gut-associated sample types and provides a resource for future comparative microbiome, functional, and antimicrobial resistance gene analyses in dairy cattle.

## ANNOUNCEMENT

Milk and fecal microbiomes are important microbial communities in dairy cattle and may serve as reservoirs of antimicrobial resistance genes in dairy production systems (1–3). However, publicly available shotgun metagenomic data sets that include both milk- and gut-associated sample types from dairy cattle in Thailand remain limited. Here, we report shotgun metagenomic sequence data generated from milk and fecal samples of dairy cattle in Thailand. This data set provides a resource for future comparative analyses of dairy cattle microbiomes, functional gene profiles, and antimicrobial resistance genes.

A total of 28 samples were included, comprising 14 composite milk samples and 14 rectal fecal samples collected from cattle on four dairy farms in Chiang Mai, Thailand. Three to four cows were sampled from each farm. Farms were selected based on accessibility, willingness to participate, and the availability of lactating dairy cattle at the time of sampling. Animals were selected based on the availability of lactating cows suitable for collection of both composite milk and rectal fecal samples. Composite milk samples were collected by pooling milk from all functional quarters of each cow. Rectal fecal samples were collected from the same animals. Sample IDs with the same number in Table 1 represent milk and rectal fecal samples collected from the same cow. All samples were transported on dry ice and stored at −80°C until DNA extraction.

**TABLE 1:** Accession numbers and sequencing summary for shotgun metagenomic sequence data from milk and fecal samples of dairy cattle in Thailand

Sample ID	BioSample	SRA accession	High-quality reads	Non-bovine reads
Milk samples (BioProject PRJNA1354576)				
01M	SAMN53042777	SRR35920863	36,141,836	693,954
02M	SAMN53042778	SRR35920862	35,543,673	1,354,145
03M	SAMN53042779	SRR35920857	43,269,428	913,346
04M	SAMN53042780	SRR35920856	35,030,671	719,179
05M	SAMN53042781	SRR35920855	37,670,647	690,091
06M	SAMN53042782	SRR35920854	37,565,909	1,079,854
07M	SAMN53042783	SRR35920853	36,837,697	911,940
08M	SAMN53042784	SRR35920852	39,629,468	1,321,718
09M	SAMN53042785	SRR35920851	47,739,652	1,204,061
10M	SAMN53042786	SRR35920850	43,542,044	2,214,169
11M	SAMN53042787	SRR35920861	34,871,644	1,172,387
12M	SAMN53042788	SRR35920860	38,772,816	2,294,963
13M	SAMN53042789	SRR35920859	38,647,503	3,183,921
14M	SAMN53042790	SRR35920858	36,620,888	1,173,797
Fecal samples (BioProject PRJNA1354578)				
01G	SAMN53043799	SRR35922066	36,502,083	36,494,996
02G	SAMN53043800	SRR35922065	37,498,601	37,455,911
03G	SAMN53043801	SRR35922060	42,254,395	42,235,008
04G	SAMN53043802	SRR35922059	34,954,015	34,931,283
05G	SAMN53043803	SRR35922058	54,738,779	54,720,343
06G	SAMN53043804	SRR35922057	36,653,952	36,646,388
07G	SAMN53043805	SRR35922056	39,156,215	39,143,154
08G	SAMN53043806	SRR35922055	35,903,115	35,793,437
09G	SAMN53043807	SRR35922054	35,363,133	35,345,954
10G	SAMN53043808	SRR35922053	36,260,410	36,218,166
11G	SAMN53043809	SRR35922064	45,585,872	45,532,957
12G	SAMN53043810	SRR35922063	36,845,307	36,824,233
13G	SAMN53043811	SRR35922062	35,400,455	35,359,990
14G	SAMN53043812	SRR35922061	35,511,164	35,481,531

Genomic DNA was extracted using the ZymoBIOMICS DNA Miniprep Kit (Zymo Research Corporation, CA, USA) according to the manufacturer’s instructions. DNA concentration and quality were assessed using NanoDrop spectrophotometry, Qubit fluorometry, and agarose gel electrophoresis. Shotgun metagenomic libraries were prepared using the VAHTS Universal Plus DNA Library Prep Kit for Illumina (Vazyme Biotech, Nanjing, China) and sequenced on an Illumina NovaSeq X platform using a paired-end 150-bp sequencing strategy.

The reads deposited in the SRA are raw demultiplexed FASTQ reads. Raw FASTQ files were processed on a high-performance computing system at the Faculty of Medicine, Chiang Mai University. Initial quality assessment was performed using FastQC v0.12.1 (4), and quality reports were summarized using MultiQC v1.27.1 (5). Adapter sequences, barcode sequences, and low-quality bases were removed using Trimmomatic v0.39 (6). Quality-filtered reads were then mapped against the *Bos taurus* reference genome GCF_002263795.3_ARS-UCD2.0 using Bowtie2 v2.5.4 (7). Reads that did not map to the bovine reference genome were retained as non-bovine reads for downstream metagenomic analyses.

The data set contains publicly available shotgun metagenomic reads from milk and fecal samples suitable for taxonomic profiling, functional profiling, and antimicrobial resistance gene detection. The retained non-bovine reads may be used to investigate microbial community composition, gene content, and resistome profiles in mammary- and gut-associated sample types from dairy cattle. This resource may also support comparative studies of microbial and antimicrobial resistance gene reservoirs in dairy production systems.

The SRA accession numbers correspond to raw demultiplexed FASTQ reads. High-quality reads indicate reads retained after quality filtering. Non-bovine reads indicate reads that did not map to the *Bos taurus* reference genome GCF_002263795.3_ARS-UCD2.0. M, milk; G, feces. Sample IDs with the same number represent milk and rectal fecal samples collected from the same cow.

## Data Availability

The raw shotgun metagenomic reads generated in this study have been deposited in the NCBI Sequence Read Archive under BioProject accession numbers PRJNA1354576 and PRJNA1354578. Milk metagenome data are available under BioProject accession number PRJNA1354576, and fecal metagenome data are available under BioProject accession number PRJNA1354578. The corresponding BioSample and SRA accession numbers are listed in Table 1.
